# LEVERAGING IOT AND NATURAL LANGUAGE PROCESSING TO IMPROVE WELL-BEING OF CHRONIC PATIENTS

**DOI:** 10.1093/geroni/igad104.2967

**Published:** 2023-12-21

**Authors:** Balakrishnan Mullachery, Samir Chatterjee, Arindam Brahma

**Affiliations:** ESRI, Claremont, California, United States; Claremont Graduate University, Claremont, California, United States; Loyola Marymount University, Aliso Viejo, California, United States

## Abstract

According to the Centers for Disease Control and Prevention, in the United States, about 80% of older adults - individuals aged 65 years and older, have at least one chronic health condition. Chronic diseases require ongoing medical attention or limit activities of daily living, which in turn, affects quality of life. Heart diseases, cancer, and diabetes are the leading chronic diseases. Literature shows that timely and frequent follow-ups by providers significantly reduce readmissions of chronic patients and contribute to their well-being. However, health care provides do not have adequate bandwidth for regular patient follow-ups, which is vital for chronic patients. One solution for this growing problem, is to leverage smart home monitoring technologies facilitating self-care, which in turn can contribute to patients’ well-being. The goal of this research is to build and leverage an IoT-based smart home monitoring platform and assess its impact on the well-being of chronic patients. The platform included an mHealth mobile app with daily journaling capability, which allowed patients to blog about their symptoms, daily activities, and feelings. Analyzing patients’ daily blog data with Natural Language Processing (NLP) methods, this research revealed a strong correlation between a patients’ blog entries and their well-being score. The results also demonstrated that patients’ well-being scores can be predicted from such journal entries analyzed at real time, collected remotely from their home environment, leading to timely diagnosis and intervention opportunities. A field trial was conducted involving five heart disease and eight cancer patients for 180 days to validate the conclusions.

